# Identification of Sequence Variation in the Apolipoprotein A2 Gene and Their Relationship with Serum High-Density Lipoprotein Cholesterol Levels

**DOI:** 10.7508/ibj.2016.02.003

**Published:** 2016-04

**Authors:** Fatemeh Bandarian, Maryam Sadat Daneshpour, Mehdi Hedayati, Mohsen Naseri, Fereidoun Azizi

**Affiliations:** 1 Diabetes Research Center, Endocrinology and Metabolism Clinical Sciences Institute, Tehran University of Medical Sciences, Tehran, Iran;; 2 Endocrinology and Metabolism Research Center, Endocrinology and Metabolism Clinical Sciences Institute, Tehran University of Medical Sciences, Tehran, Iran;; 3 Research Institute for Endocrine Sciences, Shahid Beheshti University of Medical Sciences, Tehran, Iran;; 4 Genomic Research Center, Birjand University of Medical Sciences, Birjand, Iran

## Abstract

**Background:**

Apolipoprotein A2 (APOA2) is the second major apolipoprotein of the high-density lipoprotein cholesterol (HDL-C). The study aim was to identify *APOA2* gene variation in individuals within two extreme tails of HDL-C levels and its relationship with HDL-C level.

**Methods:**

This cross-sectional survey was conducted on participants from Tehran Glucose and Lipid Study (TLGS) at Research Institute for Endocrine Sciences, Tehran, Iran from April 2012 to February 2013. In total, 79 individuals with extreme low HDL-C levels (≤5^th^ percentile for age and gender) and 63 individuals with extreme high HDL-C levels (≥95^th^ percentile for age and gender) were selected. Variants were identified using DNA amplification and direct sequencing.

**Results:**

Screen of all exons and the core promoter region of *APOA2* gene identified nine single nucleotide substitutions and one microsatellite; five of which were known and four were new variants. Of these nine variants, two were common tag single nucleotide polymorphisms (SNPs) and seven were rare SNPs. Both exonic substitutions were missense mutations and caused an amino acid change. There was a significant association between the new missense mutation (variant Chr.1:16119226, Ala98Pro) and HDL-C level.

**Conclusion:**

None of two common tag SNPs of rs6413453 and rs5082 contributes to the HDL-C trait in Iranian population, but a new missense mutation in *APOA2* in our population has a significant association with HDL-C.

## INTRODUCTION

Low high-density lipoprotein cholesterol (HDL-C) is a known risk factor of coronary artery disease (CAD) and a component of metabolic syndrome that increases the vulnerability to CAD^[^^1^^]^. The High prevalence of low HDL-C level has been reported in Iranian population^[^^2^^]^. Apolipoprotein A2 (APOA2) is the second major protein of the HDL-C particles and comprises about 20% of the total HDL-C protein content^[^^3^^,^^4^^]^.

APOA2 has antagonist effect on cellular cholesterol efflux through the modulating of the enzymes activity in HDL-C remodeling, including lecithin-cholesterol acyltransferase, cholesteryl-ester transfer protein, hepatic lipase and inhibition of lipoprotein lipase^[^^5^^,^^6^^]^. APOA2 over-expression has been shown to increase the HDL-C level in animal models, but this elevation has no protective effect on CAD^[^^7^^]^. Such evidence has not been observed in human studies. Furthermore, it has been suggested that the disorders of APOA2 metabolism could increase the risk of CAD by direct or indirect effects on serum lipids, insulin resistance, and atherosclerosis^[^^8^^]^. APOA2, which is encoded by *APOA2* gene containing three exons, has been located at chromosome 1q21-q23. The association of APOA2 variants with diabetes, obesity, and insulin resistance has been reported in the previous studies^[^^7^^,^^9^^,^^10^^]^; nevertheless, no such an association has been found for the HDL-C level. 

Because there is no data about the variants of APOA2 gene in Iranian populations with different ethnic groups, this study aimed to identify genetic variants in APOA2 gene and their association with HDL-C levels.

## MATERIALS AND METHODS

**Participants**

The present cross-sectional study was conducted on individuals with extreme HDL-C levels. The patients were selected from the participants of Tehran Glucose and Lipid Study (TLGS) at Research Institute for Endocrine Sciences, Tehran, Iran from April 2012 to February 2013. TLGS, as a family-based cohort study with more than 15,005 participants investigates the cardiovascular risk factors and disease in the residents of Tehran district 13^[^^11^^]^. All participants signed a written informed consent before enrollment, and the protocol of the study was approved by Ethics Committee at Research Institute for Endocrine Sciences at Shahid Beheshti University of Medical Sciences (Tehran, Iran). Those participants of TLGS aged between 15 and 70 years old were included in the present study. Among 10,764 individuals in these age ranges, those with extreme low HDL-C levels (≤5^th^ percentile for age and gender) and those with extreme high HDL-C levels (≥than 95^th^ percentile for age and gender) were selected. In addition, the individuals with the same trait in four TLGS phases consistently and at least a family member with the same trait were included and those with obesity (body mass index (BMI) ≥30 kg/m^2^), cigarette smoking and those receiving drugs affecting HDL-C level were excluded from the study.

Finally, 142 individuals (97 males and 45 females), including 79 individuals with very low HDL-C (59 males and 20 females) and 63 individuals with extreme high HDL-C levels (38 males and 25 females), were eligible and selected from the TLGS participants.

**Genetic analysis**

After blood sampling, Genomic DNA was isolated from peripheral blood leukocyte using proteinase K, salting-out method. Using specific primers, PCR was performed, and all three exons with at least 100-bp adjacent intronic regions were amplified. In addition to coding regions of APOA2, the regulatory region (promoter) considering at least 500 bp upstream of the transcription start site and 200 bp downstream of transcription start site located in 5’UTR was also amplified. The primer sequences used for the amplification of each APOA2 region are shown in Table 1. Specific primers were designed using Primer3 software (ver. 0.4.0). Four fragments of APOA2 gene were amplified using the following PCR protocols: 

Promoter: 94°C,10 min, 35 cycles (94°C, 50 s; 61.5°C, 45 s; 72°C, 45 s), 72°C, 10 min

Exon 1: 94°C,10 min, 35 cycles (94°C, 45 s; 52°C, 60 s; 72°C, 30 s), 72°C, 10 min 

Exon 2: 95°C, 5 min, 35 cycles (95°C 40 s; 62.5°C, 40 s; 72°C, 33 s), 72°C, 5 min 

Exon 3: 94°C, 10 min, 35 cycles (94°C 45 s; 65°C 50 s; 72°C, 30 s), 72°C, 10 min

After DNA amplification, gel electrophoresis Direct DNA sequencing on 8% polyacrylamide gel to detect exact PCR product bands. Afterwards, PCR product was sent for DNA sequencing. DNA direct sequencing was performed using an ABI Prism 3730xl DNA Analyzer (Applied Biosystems, Foster City, CA). The final obtained sequences were aligned by Chromas software (ver. 2.31), and their related electropherograms were viewed and inspected using the same software to identify single nucleotide polymorphisms (SNPs). Powermarker software version 3.25 was used to calculate the allele frequency, linkage disequilibrium (LD), and consistency with Hardy-Weinberg equilibrium for the identified variants^[^^12^^]^. The database of Genotypes and Phenotypes (dbGaP)^[^^13^^]^ as well as Ensembl Genome Browser^[^^14^^]^ were used to identify if any of the detected variants in *APOA2* gene are known SNPs and has been reported previously. Exome Variant Server database was also used to assess the functional and clinical significance of identified variants^[^^15^^]^. To predict the possible effect of an amino acid change in missense variants on the structure and function of a human protein, polyphen score was calculated using polyphen-2 software version 2.2.2^[^^16^^]^.

**Table 1:** PCR primers and related conditions for promoter and all three exons of APOA2 gene

**Segment**	**Forward primer (5** **׳** ** to 3** **׳** **)**	**Reverse primer (5** **׳** ** to 3** **׳** **)**	**Product size (bp)**
Promoter	TTTACACTTGCCCTCTTACC	CCAAATGGGCTTCAGCTC	701
Exon 1	TGTGAGCAGCATCCAAAGAG	GGTATCCAGCCAGATCTTTG	389
Exon 2	CAAAGATCTGGCTGGATACC	GTGTGGGATCTTTGGGTGAT	445
Exon 3	CGATGGACCAGGCACTAGAG	ACATACCAGGCTCAGAGCTG	372

**Table 2:** Demographic characteristics and biochemical parameters of the study participants

**Variable**	**Men** **(mean ** **±** ** SD)**	**Women** **(mean ** **±** ** SD)**	***P*** **value**
Age (year)	40.24±15.61	38.71±14.85	0.580
HDL-C level (mg/dl)	43.51±21.02	59.58±26.41	<0.001*
LDL-C level (mg/dl)	94.77±32.21	102.80±35.97	0.190
Triglyceride level (mg/dl)	181.58±18.53	116.18±86.69	<0.001*
Total cholesterol (mg/dl)	178.67±47.18	183.82±49.77	0.550
BMI (kg/m^2^)	25.60±3.51	24.92±3.44	0.270

BMI, body mass index;

* *P*<0.05 significant

**Biochemical analysis**

Biochemical parameters, including fasting blood sugar, cholesterol, triglyceride, and HDL-C were measured by enzymatic colorimetric method. LDL-C level was calculated using Friedewald’s equation^[^^17^^]^. 

**Statistical analysis**

Quantitative data were presented as mean±SD, and categorical and qualitative data were presented as number and percentage. Chi-square or Fisher’s exact test was used for the comparison of allele frequencies between the two study groups. Quantitative variables with normal distribution such as serum lipids were compared between DNA variant groups using one-way ANOVA or independent sample *t*-test as appropriate. A multiple logistic regression model was used to remove confounding factors, including age, gender, BMI, and total cholesterol, and then odds ratio (OR) was calculated. *P *values ≤0.05 were considered as significance level. All statistical analyses were carried out using SPSS software version 17.00 for Windows.

## RESULTS

**Demographics**

Due to poor quality of DNA, amplification and sequencing of DNA were carried out only on 77 individuals with extreme low HDL-C levels and 59 of those with extreme high HDL-C levels. Table 2 shows the demographic characteristics of the study participants. 

**Identified variants**

**Fig. 1:**
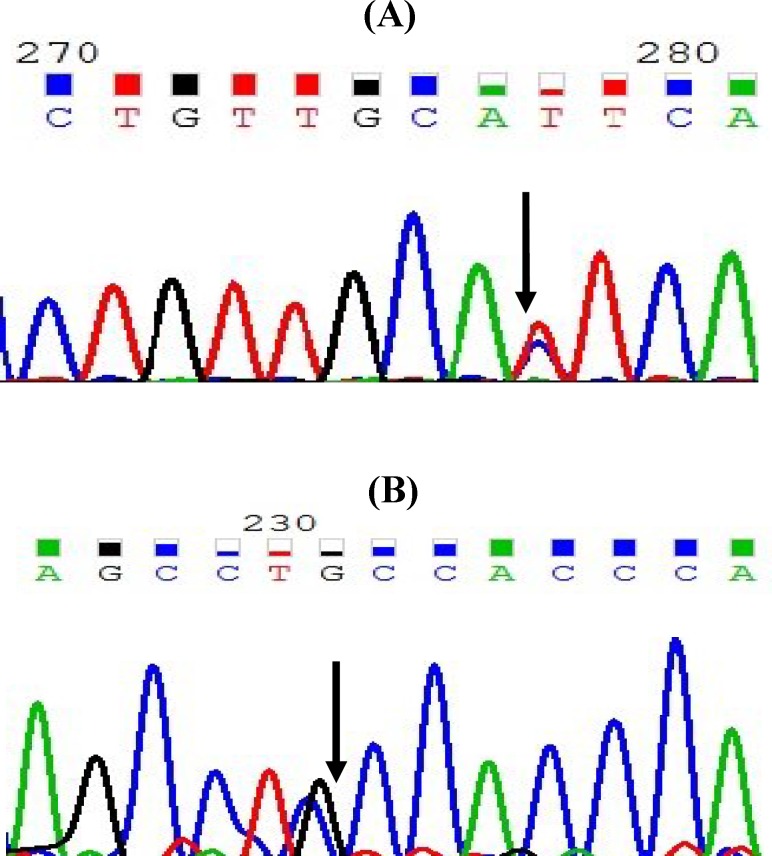
The sequence of a known (A) and new (B) variant in APOA2 sequence. Arrows show the location of variant

 After the analysis of all three exons with adjacent intronic regions and the core promoter of APOA2 in individuals with extreme HDL-C levels, nine SNPs and one microsatellite were identified (Table 3), of these variants, five had been previously reported and four were new variants (Fig. 1A and 1B). Also, three of these SNPs had been located in the regulatory (promoter) region, two in the exonic region, three in the intronic region within 100 bps of the intron/exon junction, and one in 3’UTR. The microsatellite of GT repeats was identified in intron 1, 44 bps upstream of exon 2. Two of the identified variants were common variants and others were rare SNPs. Both exonic variants were missense mutation and caused an amino acid change. Table 3 shows the summary of the identified SNPs in *APOA2* gene in individuals with extreme HDL-C levels.

**Table 3:** The identified variants of APOA2 gene in two study groups

	**Variant**	**Allele change**	**Location**	**Amino acid** **change**	**Amino acid** **position**	**Polyphen** **score**	**Allele frequency**		***P*** **value**
							**High HDL-C group**	**Low HDL-C group**	
1	rs5082 CR024268	C>T	Promoter	N/A	N/A		0.617	0.674	0.0001
2	rs12721033	G>C	Promoter	N/A	N/A		0.000	0.007	-
3	rs72661132	C>T	Promoter	N/A	N/A		0.000	0.007	-
4	rs202223831	C>G	Exon 1	Leu-Val	16	0.884 (possibly damaging)	0.000	0.006	-
5	Chr:1:16119396	G>A	Intron 1	N/A	N/A		0.000	0.006	-
6	Chr:1:1611929108	C>G	Intron 1	N/A	N/A		0.000	0.006	-
7	rs6413453 (CS900228)	C>T	Intron 2/splice region variant	N/A	N/A		0.100	0.088	0.80
8	Chr.1:16119226	G>C	Exon 3	Ala-Pro	98	0.011 (Benign)	0.036	0.000	-
9	Chr:1:161192113	C>G	3’UTR	N/A	N/A		0.000	0.007	-

N/A: not applied

**Allele frequency**

Table 3 shows the allele frequency of the identified variants in two study groups. The allele frequency of the regulatory variant of rs5082 in the extreme low HDL-C group was significantly higher than that in the extreme high HDL-C group (*P*=0.0001). All the identified variants were consistent with Hardy-Weinberg equilibrium. Linkage disequilibrium was observed between all variants that shows common inheritance patterns. Calculation of polyphen score found that one of the two missense variants to be benign and the other one possibly damaging (Table 3). 

**Associations**

There was a significant association between the new missense mutation (variant Chr.1:16119226, Ala98Pro) and the HDL-C level. Mean HDL-C concentration in those with one mutant allele (heterozygote for Ala98 Pro-variant) was significantly higher than those with two wild allele (homozygote) (*P*=0.034). The frequency of SNP Ala98Pro in high HDL-C group was significantly higher than low HDL-C group (*P=*0.024). No significant association was also found between the HDL-C level and two common variants of rs6413453 and rs5082 (*P*=0.506 and 0.346, respectively). A multiple logistic regression model was used, and confounding variables, including age, gender, cholesterol level, and BMI were entered to the model but again the association remained non-significant. 

## DISCUSSION

 In genetic screening of all exons and the core promoter of APOA2 in individuals with extreme HDL-C levels, nine variants and one microsatellite were found; Three in the promoter region, two in the coding regions, three in the intronic region adjacent to exons, and one in 3’UTR. Also, the microsatellite of GT repeats was found in the exonic region. A similar study on African Blacks and U.S. Non-Hispanic whites identified 25 variants and one microsatellite in the APOA2 gene^[^^18^^]^, which is more than our identified variants.

 This study explored *APOA2* genetic variants in two groups of extreme HDL-C levels. The study of individuals with extreme HDL-C levels increases the chance of detecting functionally significant rare and common variants with large phenotypic effects^[^^19^^]^. The higher rate of mutation in one extreme group versus the other one is a strong evidence for association between genetic variants and the specific trait^[^^19^^,^^20^^]^. By this approach, the common variants seen in both extreme groups with different frequency may have effect on the specific phenotype, whereas rare variants with a strong phenotypic effect are more likely to be found in only one extreme^[^^21^^]^.

 In this study, the frequency of common variants (rs6413453 and rs5082) was not significantly different between the two HDL-C groups. It seems that these two variants do not affect the HDL-C level. In a study conducted on 88 normolipidemic young men that were given fatty meal, it was found that the carriers of the minor allele of rs5082 (CC/TC) have a lower postprandial response compared with TT homozygotes. Its results indicated that APOA2 rs5082 may decrease the risk of cardiovascular disease due to the lower level of postprandial hypertriglyceridemia^[^^22^^]^.

 Another study on 556 Australian men with confirmed CAD and 1109 randomly selected healthy individuals showed that there is no significant association between APOA2 rs5082 and the plasma HDL-C levels, which confirms our study finding. They also concluded that this variant may be cardioprotective in their population^[^^23^^]^. In another study on 3093 French Caucasian subjects, there was no significant association between rs5082 and rs6413453 with the HDL-C level, which confirms ours. They found a marginally significant association between rs5082 and total cholesterol level as well as between rs6413453 and body weight^[^^24^^]^. 

 In line with our findings, dbGaP and Ensembl Genome Browser indicated that there is not any association between the two variants (rs5082 and rs6413453) and the HDL-C levels^[^^13^^,^^14^^].^ In contrast, Hollister^[18] ^reported a significant association between rs6413453 and the HDL-C levels in females.

The present study, for the first time, identified a new missense rare variant (variant Chr.1:16119226, Ala98Pro) that was significantly associated with HDL-C^[^^13^^,^^14^^,^^24^^]^. The main limitation of the current study was the failure of second APOA2 exon sequencing due to the presence of a long dinucleotide microsatellite of GT repeats in intron 1 and in the upstream of exon 2 that inhibited sequencing and caused incomplete sequencing. (GT)n repetitive sequences are found in the hot spot region of genome and form a left-handed helix structure (Z-DNA). Recombination rate increases in these GT repeated regions (microsatellite)^[25]^. Two other limitations of the present study are the absence of control group (normal HDL-C levels) as well as the lack of validation and genotyping of the identified variants in a large normal population. 

 This is a primary screening study, and genotyping of the identified variants will be performed in the next phase of the study by restriction fraction length polymorphism method. Nevertheless, future geno-typing studies with a large sample size in a general population are warranted to validate our study findings. In conclusion, the results do not show any contribution of two common tag SNPs of rs6413453 and rs5082 in the HDL-C trait in an Iranian population.
